# Juvenile Nasopharyngeal Angiofibroma with intradural extension

**DOI:** 10.1590/S1808-86942011000500025

**Published:** 2015-10-22

**Authors:** Henrique Faria Ramos, Marystella Tomoe Takahashi, Bernardo Faria Ramos, Marcos de Queiroz Teles Gomes, Luiz Ubirajara Sennes

**Affiliations:** 1 MD. Otorhinolaryngologist. Graduate Student - Otorhinolaryngology Program - Medical School of the University of São Paulo; 2 MD. Otorhinolaryngologist. Graduate Student - Otorhinolaryngology Program - Medical School of the University of São Paulo; 3 MD. 3rd-year resident in Otorhinolaryngology - Medical School of the University of São Paulo; 4 MD. Neurosurgeon. Assistant Physician - Neurosurgery Department - Medical School of the University of São Paulo; 5 Senior Associate Professor of Otorhinolaryngology - Medical School of the University of São Paulo

**Keywords:** angiofibroma, nasopharyngeal neoplasms, skull base neoplasms

## INTRODUCTION

The juvenile Nasopharyngeal Angiofibroma (JNA) is a benign vascular tumor of the skull base, which affects almost only male adolescents. It is a rare tumor, making up less than 0.05% of all head and neck tumors. Although histologically benign, it may have a very aggressive behavior, extending to adjacent tissues and causing bone destruction by compression^1^. The progression of such extension may cause intracranial involvement, which is relatively frequent, involving about 10% to 36% of the cases^2^. Nonetheless, it rarely goes beyond the duramater^3^,^4^.

## CASE REPORT

WLS, male, 16 years old, soccer goalie, with a past of epistaxis episodes and left nasal obstruction for 5 months; and a nasopharyngeal lesion seen upon nasal endoscopy. The CT scan showed signs suggestive of an Andrews/Fisch II^5^ JNA. In August of 2006 he was submitted to embolization of the maxillary artery, ascending pharyngeal artery and the middle meningeal; nevertheless, there still was mild tumor irrigation from the cavernous branches of the internal carotid artery. Three days later we removed the tumor endoscopically. During the surgery, the patient developed an important bradycardia upon manipulation of the pterygopalatine fossa; thus, the procedure was interrupted, with an apparent complete tumor removal.

After 1 year of follow up, the patient had symptom recurrence, and a nasopharyngeal lesion was seen upon nasal endoscopy. CT scan and MRI showed an Andrews/Fisch IVb^5^ JNA, extending to the pterygopalatine fossa, the pterygoid fossa, sphenoidal sinus, cavernous sinus and temporal lobe. Angiography showed a major vascular component coming from the petrous and cavernous branches of the internal carotid artery (ICA), after embolization of external carotid artery branches.

**Figure 1:**
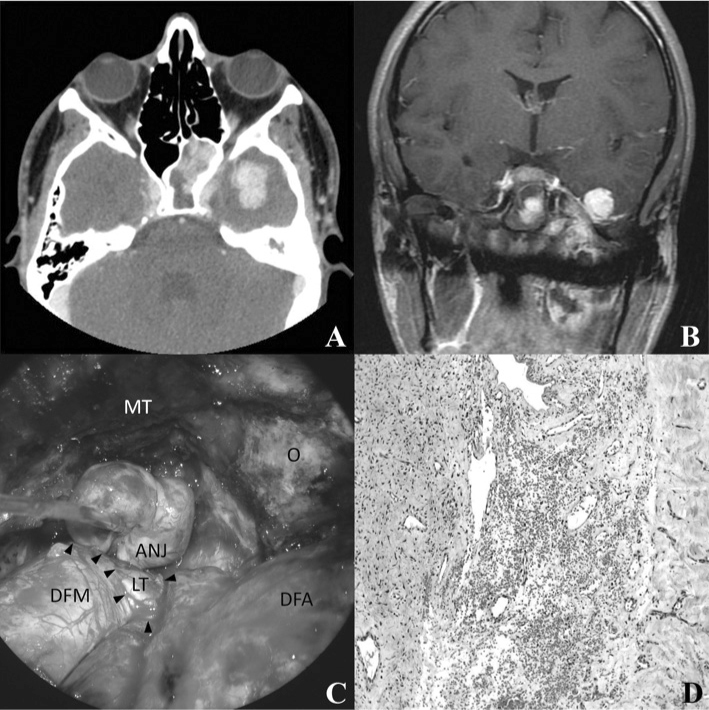
A) Axial CT showing the JNA with sphenoid sinus involvement and intradural extension, irrigated by ICA branches. B) T1-weighed coronal MRI with gadolinium showing the JNA extension to the middle fossa, in close contact with the temporal lobe. C) Microscopic view of the JNA dissection of the temporal lobe (LT) after opening the middle fossa dura mater (DFM). The arrows represent the open dura mater borders. MT. Temporal muscle; O. orbit; DFA. Anterior fossa dura mater. D) Microphotograph of the JNA fragment in the dura (left), in close contact with the dura mater (right) and tissue invasion areas (hematoxylin and eosin; 100 X)

Considering the aforementioned aspects, in October of 2007 we decided for a craniotomy approach through the left frontotemporal zygomatic via for tumor ressection, since the tumor may be detached from the ICA. Under microscopic view, the duramater was dissected from the middle fossa floor, which was opened by a high speed pneumatic burr, exposing the orbit, the round foramen with the maxillary nerve (V2), the oval foramen with the mandibular nerve (V3) and the cavernous sinus. The vascular connections between the ICA and the angiofibroma were coagulated with the bipolar cautery. Following that, we noticed that the angiofibroma was invading beyond the duramater, and after opening it we saw that the tumor was in close contact with the temporal lobe, with a clivage plane. After removing its intradural portion, the dural gap through which the tumor had passed was closed with a temporal fascia graft. The nasopharyngeal, sphenoidal and infratemporal portions were digitally dissected and removed superiorly through the middle fossa floor gap, which was later rebuilt by rotating the temporal muscle. The histopathology analysis of the intradural specimen showed a close relation between the JNA and the dura mater, with invasion of the latter (Figure 1).

The patient developed left abducent nerve paralysis in the post-operative, which lasted for 1 month. After 1 month of follow up, there were no signs of recurrence upon nasal endoscopy and MRI, or neurologic deficit, and the patient resumed his regular activities.

## DISCUSSION

JNA with skull base erosion and intracranial extension courses with an increase in operatory risks, as well as the likelihood of significant vascular contributions of the ICA, which pre-operative embolization is not feasible. For these reasons, it is associated with higher hemorrhage rates of difficult control, neurologic deficits, subtotal resection and recurrence. Previous pre-operative embolization is also associated with recurrence, since the reduction of tumors with deep invasion of the sphenoid makes it difficult to completely excise it, with quick revascularization of the residual tumor in the immediate postop, especially by ICA branches^6^.

Based on the assumption that the bone destruction mechanism by the angiofibroma results from a compressive growth pattern instead of an infiltrative one^1^, we see that intracranial extension is usually extradural^3^,^4^.

Dura penetration is a rare phenomenon, with very few reports in the literature. Upon MRI, the transdural lesion suggestive signs are an absence of the cleavage plane between the tumor and the duramater, or the circunferential involvement of the ICA; nonetheless, in some cases the MRI is unable to distinguish the invasion as extradural or intradural. The presence of colateral branches of cerebral parenchima vassels irrigating the tumor, seen upon angiography, may be a sign of duramater involvement.

Jafek et al.^2^ was the first to report on a case of JNA with dura penetration, treated by a combined otorhinolaryngological-neurosurgical approach. Lyons & Donald^7^ reported on a case of a successful surgical treatment of the JNA which penetrated the dura and the piamater of the temporal lobe. Butugan et al.^1^ reported three cases of dura transgression and cavernous sinus invasion (two of these were recurrent tumors), showing that prior manipulation predisposed the patient to dura invasion.

According to Danesi et al.^4^ the possibility of intradural extension canot be denied; nonetheless, in order to prove it, the surgeon must show, histologically, that the tumor is invading the duramater or, alternatively, see the intradural tumor through an initial neurosurgery. Having said that, the case hereby reported fills both criteria concerning intradural involvement.

## CONCLUSION

Although intracranial invasion by JNA is relatively common, its intradural extension is very rare, happening mainly in tumor recurrences. Usually the cleavage plane between the JNA and the cerebral tissue is well defined. Total tumor resection is possible with minimum neurological deficits, but dura reconstruction is necessary.
